# Immunotechniques for the Group Determination of Macrolide Antibiotics Traces in the Environment Using a Volume-Mediated Sensitivity Enhancement Strategy

**DOI:** 10.3390/bios13100921

**Published:** 2023-10-10

**Authors:** Maksim A. Burkin, Anna N. Tevyashova, Elena N. Bychkova, Artem O. Melekhin, Inna A. Galvidis

**Affiliations:** 1 I. Mechnikov Research Institute for Vaccines and Sera, 105064 Moscow, Russia; galvidis@yandex.ru; 2 Gause Institute of New Antibiotics, 199021 Moscow, Russia; chulis@mail.ru (A.N.T.); e-bychkova@mail.ru (E.N.B.); 3 School of Science, Constructor University, 28759 Bremen, Germany; 4 Department of Chemistry, Lomonosov Moscow State University, 119991 Moscow, Russia; artem150196@mail.ru; 5 Federal Centre for Animal Health, 111622 Moscow, Russia

**Keywords:** macrolide antibiotics, group recognition, hapten design, enzyme-linked immunosorbent assay, immunobeads assay, immunofiltration

## Abstract

Macrolide antibiotics, which are effective antimicrobial agents, are intensively used in human and veterinary medicine, as well as in agriculture. Consequently, they are found all over the world as environmental pollutants, causing harm to sensitive ecological communities and provoking a selection of resistant forms. A novel azithromycin derivative, which was used as hapten conjugate, ensured the group immunorecognition of six major macrolide representatives (105–41%), namely erythromycin, erythromycin ethylsuccinate, clarithromycin, roxithromycin, azithromycin, and dirithromycin in a competitive immunoassay based on anti-clarithromycin antibodies. The heterologous hapten-based ELISA format resulted in a 5-fold increase in sensitivity, with an IC_50_ value of 0.04 ng/mL for erythromycin. In this study, we proposed an underexploited strategy in an immunoassay field to significantly improve the detectability of analytes in environmental samples. Unlike most approaches, it does not require special enhancers/amplifiers or additional concentration/extraction procedures; instead, it involves analyzing a larger volume of test samples. A gradual volume increase in the samples (from 0.025 to 10 mL) analyzed using a direct competitive ELISA, immunobeads, and immunofiltration assay formats based on the same reagents resulted in a significant improvement (more than 50-fold) in assay sensitivity and detection limit up to 5 and 1 pg/mL, respectively. The suitability of the test for detecting the macrolide contamination of natural water was confirmed by the recovery of macrolides from spiked blank samples (71.7–141.3%). During 2022–2023, a series of natural water samples from Lake Onega and its influents near Petrozavodsk were analyzed, using both the developed immunoassay and HPLC-MS/MS. The results revealed no contamination of macrolide antibiotic.

## 1. Introduction

Macrolide antibiotics are a family of drugs united by a similar structure, consisting of a 14–16-atom macrocyclic lactone ring with carbohydrate substituents [1]. Here we consider 14- and 15-membered erythromycin-based cousins, which all carry desosamine and cladinose/oleandrose linked by a glycosidic bond (Figure S1), unlike 16-membered macrolides having distinct sugar moieties (mycinose, mycaminose, and mycarose) [2].

**Figure 1:**
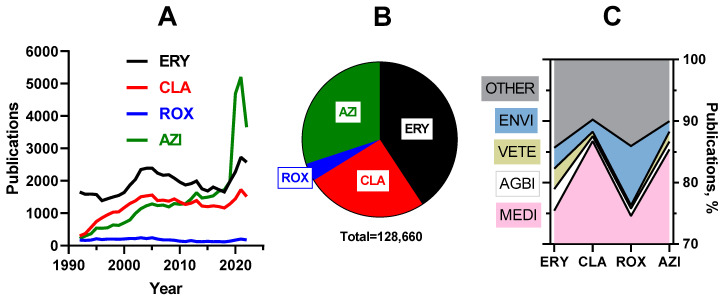
(**A**) Scopus database publications (1992–2022) by macrolide antibiotics (‘erythromycin’ OR ‘clarithromycin’ OR ‘roxithromycin’ OR ‘azithromycin’ mentioned in title, abstract, or keywords). (**B**) The share of publications of each representative of macrolides. (**C**) Distribution of publications by subject MEDI (medicine), AGBI (agricultural and biological sciences), VETE (veterinary), ENVI (environmental science), and OTHER areas.

Erythromycin (ERY) and oleandomycin (OLE) are the very first natural representatives of macrolide antibiotics, which have been isolated and used since 1952/1954. Esters of ERY and semi-synthetic derivatives, such as dirithromycin (DIR), clarithromycin (CLA), roxithromycin (ROX), and azithromycin (AZI), which are more stable in an acidic environment than ERY, date back to the 1980s [3]. Currently, OLE is not used in practice as monotherapy but is registered as a drug combination with tetracycline (Oletetrin^TM^). DIR is not manufactured in Russia and the United States; however, it is still available in many European countries. Tulathromycin (TUL) is a veterinary antibiotic indicated only for usage in cattle, pigs, and sheep [4]. Each of the three macrolides—OLE, DIR, and TUL—were mentioned in less than 1% of publications and, therefore, those were not included in the following charts. The main representatives of the macrolides in scientific research are ERY, CLA, AZI, and ROX (Figure 1).

Interest in AZI increased significantly during the SARS-CoV-2 pandemic (Figure 1A). In addition to its efficiency against sensitive bacterial co-infection, AZI has demonstrated in vitro activity against SARS-CoV-2 virus and can act at various stages of the viral cycle. Its immunomodulatory properties, and ability to suppress cytokine production, has been associated with reduced mortality and ventilator days in some studies [5]. Overall, a bibliographic search demonstrates that over the past 30 years, the scientific literature on 14- and 15-membered macrolides has been mainly devoted to the following areas: ERI (46.3%), AZI (34.2%), and CLA + ROX (29% + 4.2%) (Figure 1B). 

The results of the subject area queries indicate that the vast majority (75–85%) of research is related to the field of medicine, which is the main sphere of macrolide application. Scientific focus on the veterinary and agrobiological use of macrolides is primarily centered on ERY, which is approved for farm animals and accounts for 3.3% and 3.5% of all ERY publications, respectively. The share of human antibiotics CLA, ROX, and AZI in these areas is more modest, at around 1% each (Figure 1C). It is worth highlighting the high share (10%) of environmental studies among ROX-queried publications. However, the absolute number of these ROX studies is comparable to those for CLA and AZI. Meanwhile, ERY’s impact on environmental research is as strong as that of CLA, ROX, and AZI combined, due to its long-standing use in both human and veterinary medicine.

Indeed, all the mentioned macrolides are commonly found as contaminants in various aquatic environments worldwide with concentrations from ng/L to µg/L [6,7,8,9]. The effect of macrolide exposure on the growth, metabolism, antioxidant system, photosynthesis, DNA replication, and repair in the eco-community of algae, viruses, bacteria, crustaceans, invertebrates, and fish has been noted in many studies [10,11,12,13]. Therefore, as awareness of the potential harm of antibiotic residues to aquatic organisms increases, several antibiotics, including ERY, CLA, and AZI, have been placed on the European Union (EU) watch list of new water pollutants [14].

Environmental pollution monitoring requires particularly highly sensitive methods capable of detecting trace amounts of pollutants, which are diluted multiple times in the environment and present at very low concentrations. In such cases, additional enrichment and preconcentration of the test sample become essential for sample preparation [9,15,16,17]. 

Accordingly, the current study aims to enhance the group specificity of the immunochemical method for the detection of key macrolide antibiotics as frequent water pollutants using a novel hapten design, and to develop an approach to detect trace amounts of analytes by involving a larger sample volume without additional concentration/extraction procedures. 

## 2. Methods

### 2.1. Chemicals and Reagents

Erythromycin (ERY), erythromycin ethyl succinate (ESE), clarithromycin (CLA), roxithromycin (ROX), azithromycin (AZI), tulathromycin (TUL), oleandomycin (OLE), 1,2-ethylenediamine (EDA), 1-ethyl-3-(3-dimethylaminopropyl) carbodiimide (EDC), N,N′-dicyclohexylcarbodiimide (DCC), N-hydroxysuccinimide (NHS), carboxymethoxylamine hemihydrochloride (CMO), sodium periodate (PI), sodium borohydride, dimethyl adipimidate (DMA), bovine serum albumin (BSA), gelatin (GEL), and horseradish peroxidase (HRP) were purchased from Chimmed (Moscow, Russia). Goat anti-rabbit IgG antibodies conjugated to horseradish peroxidase (GAR-HRP) were purchased from Imtek Ltd. (Moscow, Russia). Dimethylformamide (DMF), glutaraldehyde (GA), and ovalbumin (OVA) were obtained from Serva (Heidelberg, Germany). 9-(Carboxymethyloxime)-clarithromycin (cmoCLA) and conjugated antigens and antibodies against BSA-cmoCLA(ae) were prepared and described in our previous work [18].

### 2.2. Hapten Synthesis

TLC analysis was performed on the Silica gel 60 F254 plates (aluminum sheets 20 × 20 cm) Merck (Darmstadt, Germany). Compounds were purified to have purity higher than 90% by normal phase flash or column chromatography on Merck silica gel (0.040–0.063 mm) (Darmstadt, Germany) or crystallization. The purity was assessed by reverse phase HPLC, which was carried out on a Shimadzu HPLC instrument of the LC 10 series (Kyoto, Japan) on a Kromasil-100 C18 column (4.6 × 250 mm, particle size 5 µm, Ekzo Nobel, Goteborg, Sweden) with an injection volume of 20 µL (concentration of substances 0.25–0.5 mg/mL) at a flow rate of 1.0 mL/min and was monitored by a diode array ultraviolet detector at 280 nm. The system consisted of buffer—0.2% HCOONH_4_ at pH 4.2—and organic phase—acetonitrile. The proportion of acetonitrile varied from 20 to 80% for 30 min. ^1^H and ^13^C-NMR spectra were recorded at 30 °C on a Bruker 400 NMR spectrometer at 400 and 100 MHz, respectively. Chemical shifts are expressed in δ ppm referenced to an internal tetramethylsilane (δ = 0 ppm) standard. Abbreviations used in describing peak signals are br = broad, s = singlet, d = doublet, dd = doublet of doublets, t = triplet, q = quartet, m = multiplet. ESI MS spectra were recorded on a Bruker microTOF-Q II™ instrument (BrukerDaltonics GmbH, Bremen, Germany).

**aecAZI.** *11,12-cyclic carbonate of azithromycin* (**2**). Ethylene carbonate (4 g, 45.5 mmol) was added portion-wise to a stirred solution of AZI (6 g, 8.02 mmol) and K_2_CO_3_ (1.6 g, 11.58 mmol) in ethyl acetate; the reaction mixture refluxed for 24 h and then concentrated in vacuo. CHCl_3_ (100 mL) and H_2_O (100 mL) were added to the residue. The water fraction was extracted with CHCl_3_ (2 × 50 mL). The combined organic layers were washed with H_2_O (2 × 100 mL), dried over Na_2_SO_4_, and evaporated to dryness to give target compound **2** as a white solid. Yield: 5.9 g (95%). R_f_ = 0.25 (CHCl_3_/CH_3_OH, 6:1); mp 140–142 °C; MS (ESI) *m*/*z* calculated for C_39_H_70_N_2_O_13_ 774.4878; found (M + H)^+^ 775.4824.

*2′-O-Acetyl 11, 12-cyclic carbonate of azithromycin* (**3**). Acetic anhydride (0.5 mL, 5.34 mmol) and Et_3_N (1.48 mL, 10.68 mmol) were added to a solution of 11, 12-cyclic carbonate of azithromycin (**2**, 2.0 g, 2.67 mmol) in CH_2_Cl_2_ (20 mL). The reaction mixture was stirred at room temperature for 24 h, then 5% aqueous solution of NaHCO_3_ (20 mL) was added, and the water layer was extracted CH_2_Cl_2_ (2 × 10 mL). Combined organic layers were washed with H_2_O (2 × 10 mL), dried over Na_2_SO_4_, and concentered in vacuo. The residue was purified using the flash chromatography method on silica gel (CH_2_Cl_2_/CH_3_OH, 10:1). Fractions containing the target compound were combined and evaporated in vacuo to dryness to give target compound **3** as a white foam solid. Yield: 1.5 g (75%). R_f_ = 0.6 (CHCl_3_/CH_3_OH, 6:1); mp 134–136 °C; MS (ESI) *m*/*z* calculated for C_41_H_72_N_2_O_14_ 816.4984; found (M + H)^+^ 817.5067. 

*11-O-(2-aminoethyl)carbamoyl azithromycin* (**4**). 2′-*O*-Acetyl 11,12-cyclic carbonate of azithromycin (**3**, 1.0 g, 1.22 mmol) was dissolved in EDA (4 mL), and pyridine hydrochloride (140 mg, 1.22 mmol) was added. The reaction mixture was stirred at room temperature for 48 h, and then ethyl acetate (50 mL) and H_2_O (40 mL) were added. The organic layer was separately extracted ethyl acetate (2 × 20 mL). Combined organic layers were washed with H_2_O (2 × 40 mL), dried over Na_2_SO_4_, and concentered in vacuo. The residue was purified using the flash chromatography method on silica gel (CH_2_Cl_2_/CH_3_OH, 10:1). Fractions containing a target compound were combined and evaporated in vacuo to dryness to give target compound **4** (aecAZI) as a light foam. Yield: 0.817 g (80%); R_f_ = 0.15 (CHCl_3_/CH_3_OH/NH_3_, 3:1:0.1); MS (ESI) *m*/*z* calculated for C_41_H_78_N_4_O_13_ 834.5565 found (M+H)^+^ 835.7054. R_t_ 5.05 min. ^1^H NMR (400 MHz, CDCl_3_, δ ppm): 5.02 (s, 2H), 4.91 (d, 1H), 4.49 (d, 1H), 4.41 (d, 1H), 4.30 (s, 1H), 4.10 (t, 1H), 3.29–3.32 (m, 4H), 3.13 (t, 3H), 3.02 (d, 1H), 2.35 (m, 7H), 2.23 (m, 3H), 1.98 (m, 3H), 1.73–1.76 (m, 1H), 1.66 (t, 1H), 1.55 (m, 2H), 1.36–1.17 (m, 15H), 1.13 (m, 2H), 1.09 (d, 3H), 0.99 (d, 2H), 0.86 (t, 2H). ^13^C NMR (100 MHz, CDCl_3_, δ ppm): 177.1, 158.1, 104.2, 96.2, 78.8, 78.1, 75.3, 74.3, 73.1, 70.9, 70.7, 66.1, 65.8, 62.2, 62.1, 49.8, 45.9, 43.1, 42.4, 40.7, 39.7, 37.8, 36.2, 35.2, 31.7, 29.9, 29.8, 29.7, 29.6, 29.5, 29.4, 27.2, 22.9, 22.3, 21.82, 21.5, 18.2, 14.8, 11.6, 10.7, 10.2. 

**cmoERY.** CMO-derivative of ERY was synthesized according to the procedure described elsewhere [19]. Briefly, CMO (10 mg, 78 µmol) was dissolved in 2 mL of water and added dropwise to ERY solution (20 mg, 27 µmol) in 2 mL of ethanol. The pH was adjusted to 5.5 by using a 1M NaHCO_3_. The mixture was incubated for 5 h at 50 °C and then cooled to room temperature. To extract cmoERY, CH_2_Cl_2_ (5 mL) was added. The organic phase was evaporated in vacuo; a brown oily residue was dried out using Na_2_SO_4_ and confirmed by HPLC-MS/MS. 

### 2.3. Preparation of Coating Antigens

**GEL-cmoCLA(ae).** Cmo-CLA (5 mg, 6.1 µmol) in 1 mL of DMF was supplemented with NHS and EDC (10 µmol) from 10 mg/mL solution in DMF). After stirring a mixture for 1.5 h, the activated cmo-CLA was dropwise added to GEL (8 mg, 50 nmol) in 1 mL of carbonate–bicarbonate buffer (CBB, 0.05 M, pH 9.5) and stirred overnight at room temperature. Molar ratios between protein and hapten were taken as 1/10 and 1/30. 

**OVA-cmoERY(ae).** Cmo-ERY (8 mg, 10 µmol) was dissolved in 1 mL DMF and supplemented with DCC (4 mg, 20 mmol) and NHS (2.3 mg, 20 mmol). The mixture was stirred for 4 h; after that some precipitated DCC-urea was removed by centrifugation. Then, the activated cmo-ERY was dropwise added to OVA (4.5 mg, 100 nmol) in 2 mL of water, stirred using a magnet stirrer, and kept at 4 °C overnight. 

**OVA-aecAZI(ga).** Mixtures containing 3,6 mg of OVA (80 nmol) and a 10- or 30-fold molar excess of aecAZI (0.67 and 2.0 mg, respectively) in 1 mL of CBB were composed. Freshly prepared 2.5% glutaraldehyde solution (40 µL, 10 µmol) were added to each mixture and stirred for 2.5 h using a magnet stirrer. And extra 1 h of stirring was conducted after the addition of 100 µL of sodium borohydride (1.9 mg/mL). 

**OVA-aecAZI(dma).** The mixtures containing 3.6 mg of OVA (80 nmol) and 10- or 30-fold molar excess of aecAZI (0.67 and 2.0 mg, respectively) in 1 mL of CBB were supplemented with DMA (2.45 mg, 10 µmol) in 100 µL of CBB and stirred for 2.5 h.

**OVA(pi)-aecAZI.** OVA (9.0 mg, 200 nmol) in 1 mL of 10 mM acetic buffer (pH 5.0) was supplemented with sodium periodate (2.14 mg, 10 µmol) from 10 mg/mL solution and stirred for 20 min. After oxidation, excessive reagents were removed via overnight dialysis against 5 L 10 mM acetic buffer. The volume of dialysate was measured, and portions of oxidized OVA (3.6 mg, 80 nmol) were added to solutions of 0.67 or 2.0 mg aecAZI (10- and 30-fold molar excess over OVA, respectively) in 0.5 mL CBB and stirred 2.5 h at room temperature. To stabilize conjugates, 100 µL of sodium borohydride (1.9 mg/mL) was added to each reaction mixture and stirred for 1 h.

To remove the unreacted low-molecular-weight ingredients, the resultant conjugates were dialyzed using Visking tubes (Sigma, St. Louis, MO, USA, MWCO 14 kDa) against 2 × 5 L of 0.9% NaCl, pH 7.4 for 48 h. The dialysates were supplemented with glycerol and stored as 1 mg/mL solutions at −20 °C until use. 

### 2.4. Indirect Competitive Enzyme-Linked Immunosorbent Assay (icELISA)

The general ELISA procedure, buffers, washing steps, temperature and duration of incubations, registration, and the processing of results did not differ from [20]. In present work, we investigated and compared several new-designed and previously established coating antigens, which were adsorbed on the 96-well Costar plates from 0.1 to 3.0 µg/mL solutions in CBB (pH 9.6) overnight at 4 °C. The number of analytes, macrolides to be analyzed as cross-reactive substances were expanded in this work and represented by CLA, ERY, ESE, ROX, AZI, DIR, TUL, and OLE. Solutions of these analytes (1 pg/mL–1 μg/mL) were added to wells of the plate along with anti-cmoCLA antibody in PBS-T with 1%BSA and incubated for 1 h at 25 °C in plate-shaker ST-3 L (ELMI Ltd. laboratory equipment, Riga, Latvia). GAR-HRP was used to detect antigen-antibody formed complexes for 1 h at 37 °C. The activity of the bound enzyme was detected using TMB-substrate mixture, and the intensity of colored product was read at 450 nm using a LisaScan spectrophotometer (Erba Mannheim, Czech Republic). Antibody-binding absorbance was maximal at a zero concentration of cross-reactant (B_0_) and was dose-dependently inhibited by cross-reactant. The relative antibody-binding absorbance (B/B_0_) was determined for each concentration of analyte and used to construct a calibration curve as B/B_0_ versus the concentration. Structurally related macrolides CLA, ESE, ROX, AZI, DIR, TUL, and OLE were analyzed for their cross-reactions. The concentrations resulting in half-maximal absorbance (IC_50_) served for the determination of cross-reactivity (CR = 100% × IC_50 ERY_/IC_50 ANALOG_). Assay sensitivity, limit of detection (LOD), and working range of the assay were set as values of IC_50_, IC_10_, and IC_20_-IC_80_ range, respectively. 

### 2.5. HRP-Labeled Antigen Preparation

Hapten conjugation to HRP was conducted using the periodate method as in a previous work [21]. Briefly, HRP solution (3.2 mg, 80 nmol) in 0.4 mL H_2_O was combined with an equal volume of sodium periodate (1.7 mg, 8 µmol) and stirred for 15 min with a magnet stirrer. Overnight dialysis against 0.01 M acetic buffer (pH 4.5) was conducted after to remove excess periodate. The oxidized HRP was added dropwise to aecAZI dissolved in CBB (pH 9.6) and stirred for 2 h at RT. The molar ratio between hapten and enzyme was taken in coupling as 1:3 and 1:10. To reduce the resulting Schiff base, 50 µL of an aqueous solution of sodium borohydride (2 mg/mL) was added and stirred for 1 h. After dialysis, HRP-aecAZI stabilized with 1%BSA-PBS in 50% glycerol was stored at −20 °C until use.

### 2.6. Immunosorbent Preparation

Sepharose 4B or Sepharose-CL-2B (Pharmacia, Uppsala, Sweden) were washed with water on a porous glass filter and then squeezed out with a soft press from excess moisture. The washed beads (2 g) were placed in a vial with sodium periodate solution (60 mg in 5 mL H_2_O) and mixed with a rotary mixer (ELMI Ltd. laboratory equipment, Riga, Latvia) for 45 min. An additional 30 min rotation continued after the addition of ethylene glycol (250 µL). The beads were then washed with water and finally with CBB (pH 9.6) squeezed mildly out. Each of the activated sorbents was placed in a vial for mixing with anti-cmoCLA IgG (10 mg) in 2 mL CBB (pH 9.6) for 48 h at 4 °C. The excess antibody was squeezed out. The beads were then washed with water and placed in a sodium borohydride solution (4 mg/4 mL) for 1 h with occasional shaking. After the final washing with water and PSBT, the resulting immune sorbents were stored at 4 °C in 10 mL of PSBT preserved with Merthiolate (1 mg).

### 2.7. Direct Competitive Assay Formats

To implement these assay formats, the IgG fraction was first isolated from antiserum via the double precipitation method using caprylic acid and ammonium sulfate, described in detail in [22]. The principle of the assays is based on competition between analyte and enzyme-labeled antigen (HRP-aecAZI) for binding to an antibody coated on the plate (dcELISA) or coupled covalently to Sepharose beads. The latter immunosorbent was used in the immunobeads assay (IBA) and immunofiltration assay (IFA).

**dcELISA plate format.** The direct competitive antibody-coated ELISA format was carried out in accordance with the generally accepted procedure. The antibody coated on polystyrene plates could capture the free analyte from the tested sample and enzyme-labeled hapten in a competitive manner. The sequence of manipulations corresponded to those described for a similar format earlier [21]. The role of the volume ratios between the standard (25–275 µL) and HRP-aecAZI (25–175 µL) on the sensitivity of the assay was studied in this work.

**Immunobead assay (IBA).** Standard solution of analyte or tested sample in 1–2 mL PBST were accomplished with HRP-aecAZI and immunosorbents Anti-CLA(IgG)-S4B or Anti-CLA(IgG)-SCL2B taken in optimized concentration/volume and incubated for 0.5–3 h periods at a rotary mixer mixing (15 rpm). The beads were then pelleted using centrifugation (5 min, 3400× *g*) and suspended to the original volume with PBST. After three washing cycles, the bead pellet was suspended with TMB-substrate mixture (200 μL), and 8 M sulfuric acid (50 μL) was added 30 min later to terminate enzymatic reaction. The intensity of the colored product was measured using a plate reader at 450 nm.

**Immunofiltration assay (IFA).** The IFA principle consisted of passing a mixture of standard/sample (10 mL) and HRP-aecAZI through the beads with immobilized antibodies (Anti-CLA(IgG)-S4B or Anti-CLA(IgG)-SCL2B) placed in a homemade column and recording the enzymatic activity of the captured HRP-aecAZI. Briefly, the optimal volume of the prepared immunosorbent was placed on a pre-moistened filter support in a 10 μL filtered micropipette tip, which was then accurately attached to a syringe. This homemade column was filled with 10 mL of the standard analyte or water sample, avoiding the formation of bubbles. The standards/samples were allowed to drip freely through the tips, or external pressure was applied using a peristaltic pump. A washing of beads with 3–5 mL of PBST was followed. Then, the ends of the tips were cut off, and filter pads with a layer of sorbent were pushed/washed out of the tips with a reverse flow of 200 µL of the TMB-substrate mixture into the wells of the plate. The enzymatic reaction was terminated, and the optical signal was registered as above.

### 2.8. Sample Pretreatment and Analysis

Samples of tap, natural, or waste water were filtered through a paper filter to eliminate foreign inclusions if necessary. Then, to minimize differences in pH, ionic strength, and possible non-specific interactions, the samples were supplemented with 25xPBST-concentrate and analyzed for traces of macrolides using a developed immunoassay. Parallelly, the samples were tested using HPLC-MS/MS to verify macrolide type, including 16-membered macrolides (Table S1, Figure S2). The procedure employed is described in the Supplementary Information.

A blank natural water sample verified using HPLC-MS/MS was spiked with macrolide analytes, pretreated as above, and tested in the developed immunoassay to determine recovery. 

## 3. Results and Discussion

### 3.1. Synthesis of Haptens

**Scheme 1:**
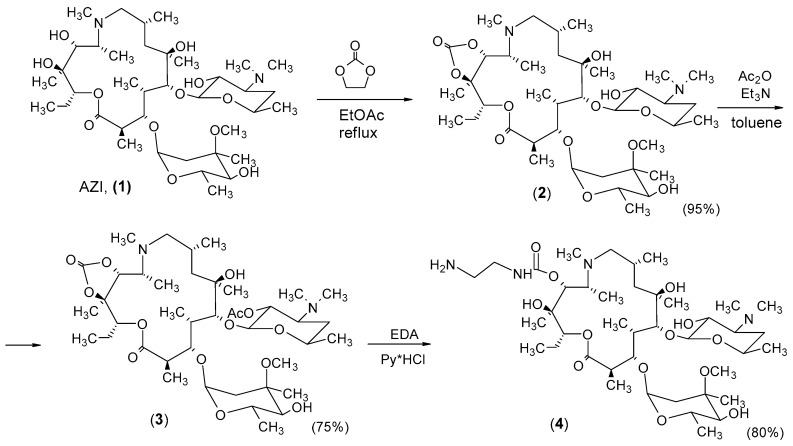
11-*O*-(2-aminoethyl) carbamoyl derivative of azithromycin (aecAZI) synthesis.

The method described by S. Ma et al. [23] and S. Printsevskaya et al. [24] was adopted for the synthesis of the target 11-O-aminoethylcabamoyl derivative of azithromycin (**4**) (Scheme 1). 

The reaction of AZI with ethylene carbonate resulted in the formation of the 11,12-cyclic carbamate of azithromycin (**2**); the 2′-hydroxylic group was protected by the acetyl group by the reaction of compound **2** with acetic anhydride in the presence of trimethylamine. The opening of the 11,12-cyclic carbonate ring of the resulting compound **3** was carried out in the EDA used as a solvent. This reaction took place in the presence of pyridine hydrochloride at room temperature, resulting in the desired 11-O-aminoethylcabamoyl derivative of azithromycin (**4**).

The purity of the compound was determined by TLC and HPLC; the structure was confirmed using HR-ESI mass-spectrometry and ^1^H and ^13^C NMR spectra. The assignment of signals in the ^1^H and ^13^C NMR spectra was made using the described assignments of the signals in the ^13^C NMR of azithromycin [25,26]. ^13^C NMR spectrum of compound **4** contains all the signals of carbon atoms corresponding to the azithromycin residue; additionally, signals corresponding to the introduced carbon atoms were observed at 158.1 ppm (corresponding to the carbamoyl carbon atom), and two signals were observed at 25–34 ppm (corresponding to the two aliphatic carbon atoms of the spacer residue).

### 3.2. Preparation of Conjugated Antigens

Along with the homologous GEL-cmoCLA(ae), a number of heterologous conjugates were obtained and investigated as coating antigens in this work. As indicated in our previous study, the main target epitopes of antibody response against BSA-cmoCLA(ae) were carbohydrate fragments, L-cladinose, and D-desosamine, which ensured the group recognition of macrolides [18]. Nevertheless, 15-membered macrolide, AZI with the same carbohydrate substituents, exhibited weaker recognition (CR = 8.8%) in comparison with analogues ERY, CLA, and ROX (68–125%). However, AZI has gained significant popularity in recent years due to the SARS-CoV-2 pandemic. This has provided an additional incentive to improve AZI recognition to the level of other members of the macrolide group.

**Figure 2:**
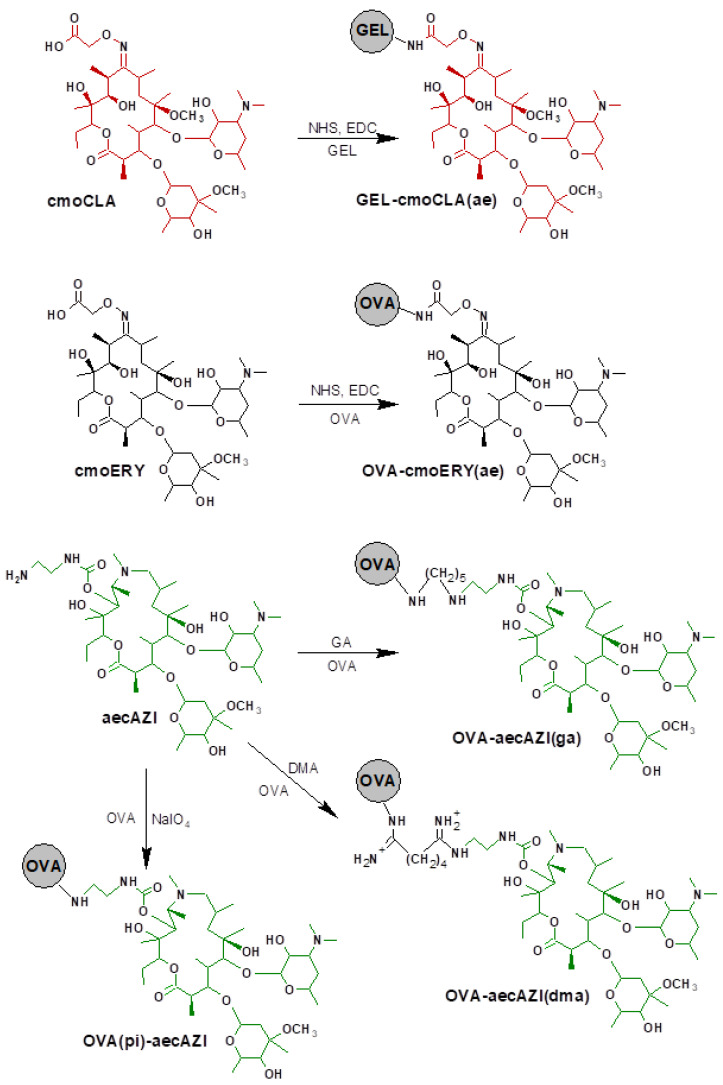
Preparation of coating conjugates.

The literature search on the competitive immunoassay of small analytes has shown that an effective way to shift the cross-reactivity profile towards the desired cross-reactant is to use the latter as a coating or a labeled hapten. The phenomenon of immunorecognition enhancement was clearly observed with such analytes as spiramycin, vancomycin [27], and teicoplanin [28]. As can be seen from Figure 2, five coating conjugates were prepared. These conjugates were designed to introduce a novel hapten molecule without affecting the target carbohydrate determinants to ensure the safety of common epitopes.

Thus, along with homologous GEL-cmoCLA(ae), heterologous OVA-cmoERY(ae), and several heterologous conjugates based on AZI-derivative, were synthetized to study their effect on assay specificity.

### 3.3. Specificity and Sensitivity of the Developed ELISA Variants

The present attempt to improve the recognition of AZI, a macrolide with a 15-membered macrocycle, involved modifying the design of the coating hapten and testing the specificity of the respective ELISA variants (Table 1). Furthermore, the detectability of four additional macrolides (ESE, DIR, TUL, and OLE) was also examined.

The initial specificity study indicated that the target epitopes for antibodies are the carbohydrate fragments of macrolides [18]. The current cross-reactivity analysis suggests that the primary target epitope is cladinose, as its modification in OLE and TUL (Figure S1) makes them poor cross-reactants. At the same time, the presence of a large substituent, ethyl succinate, in ESE’s desosamine was not critical for recognition by antibodies.

In the coated OVA-cmoERY(ae), ERY as a heterologous hapten exhibited minimal differences from the immunizing hapten, resulting in a negligible impact on the profile of cross-interactions. Similar cross-reactivity (CR) and sensitivity (IC_50_) values were obtained in this assay variant for CLA, ERY, ROX, and AZI when compared to the homologous GEL-cmoCLA(ae) [18]. 

Additionally, ESE (37%) and DIR (31%) were added to the list of recognized analytes, while TUL and OLE displayed relatively weak inhibitory effects (2.7% and 0.2%, respectively). All ELISAs utilizing coated AZI conjugates demonstrated better AZI detectability (13–41%) compared to the initial two assays (9%). The presentation of a heterologous 15-membered AZI ring through a long spacer in the form of 5/6-atom chains (ga/dma) for antibody recognition had a similar and moderate effect on cross-reactivity (13.7% and 12.9%). However, zero-length conjugation at OVA(pi)-aecAZI led to masking of the distinctive features of the 15-membered macrocycle and increased cross-reactivity with AZI up to 41%.

Comparative cross-reactivity examinations between assay variants revealed that the OVA(pi)-aecAZI-ELISA was characterized by the best group detection of six macrolides (CLA, ERY, ESE, ROX, AZI, and DIR), with differences of only 2.6 times (105–41%). In summary, the newly designed heterologous hapten conjugate improved assay sensitivity by four times and increased the detectability of AZI from 9% to 41%.

### 3.4. Direct Competitive Assay Formats

As a result of interactions between antibody and a number of coating antigens, we have identified a preferred hapten and a rational antigen design for the most sensitive and broad-specific detection. Since the aecAZI hapten conjugated to the carrier via a zero-length arm yielded the desired assay performance, we prepared the same design enzyme-labeled hapten for the direct competitive assay format. Consistent with our previous observation [21], binding an enzyme-labeled hapten with a lower molar ratio between hapten and HRP (3:1) resulted in better analyte competition and assay sensitivity compared to a 10:1 ratio. Thus, a direct competitive assay format was optimized and established based on coated anti-BSA-cmoCLA(ae) antibody and HRP-aecAZI × 3 as a tracer.

It is a common practice to express and compare the characteristics of methods in concentration units. This is because the assay protocol typically involves the use of fixed sample volumes. However, the actual factor is the amount of analyte in the analysis. In this study, we examined the effect of varying the sample volume on assay sensitivity in an effort to improve assay performance using the internal resource of available reagents without the need for additional complicating approaches, special enhancers, or signal amplifiers.

### 3.5. Effect of Sample Volume Increase on Sensitivity in Plate Assay Format

**Figure 3:**
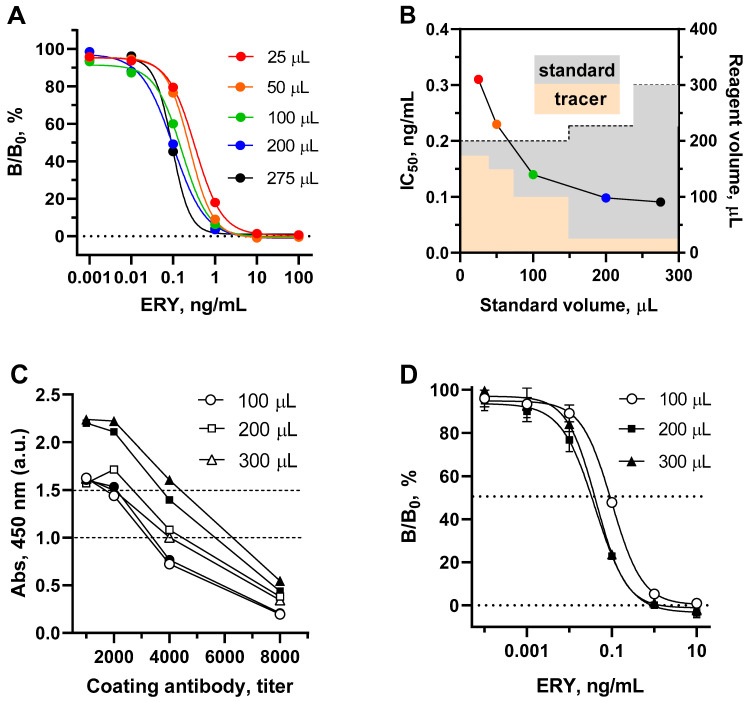
(**A**) Effect of standard and reagents volume in the well on direct competitive ELISA calibration curve. (**B**) Dependence of ELISA sensitivity on immunoreagent volumes. The ratios of standard/tracer HRP(pi)-aecAZI volumes were following 25/175 µL, 50/150 µL, 100/100 µL, 200/25 µL, and 275/25 µL. Tracer concentrations were 0.57-, 0.67-, 1-, 4-, and 4-fold of working concentration, respectively. (**C**) Optimization of coating antibody volume (100–300 µL) and concentration (1/1000–1/8000). Substrate/stop solution volumes were 100/100 µL (empty symbols) and 200/50 µL (filled symbols). The optimal absorbance range of 1.0–1.5, indicated by dotted lines, served to determine the working antibody titer. (**D**) Effect of coating antibody and substrate volumes on ELISA signal and sensitivity. Antibody (4.3 mg IgG/mL) was coated at 100 µL, 200 µL, and 300 µL per well from 1/2000, 1/4000, and 1/5000 solutions, respectively. The ratio of standard/tracer HRP(pi)-aecAZI volumes was 275/25 µL. Substrate/stop solution volumes were 100/100 µL (empty symbols) and 200/50 µL (fill symbols).

In the plate assay format, reaction volumes are limited to a well volume of 350–400 μL, allowing flexibility in adjusting the ratio of reactant volumes within this range. Consequently, the standard curves for ERY, used as a model analyte, clearly shift to the left as the volume of the analyte increases (Figure 3A).

To address this, the volume of the tracer was reduced by proportionally increasing its concentration, ensuring that the total volume of the reaction mixture did not exceed 300 μL, as shown in Figure 3B. Increasing the volume of the ERY standard in this experiment from 25 to 275 µL resulted in an improvement in assay sensitivity (IC_50_) from 0.31 to 0.091 ng/mL.

Since the volume of competitive interaction reaches 300 µL, it is logical that the well surface coated with antibody should be broadened appropriately. To achieve this, the coating antibody concentration, as well as the volumes of substrate and stop solution volumes, were optimized (Figure 3C).

It can be observed that the optical signal increases with an increase in the surface of the well coated with antibody. This growth was particularly pronounced when the volume of the substrate was also increased from 100 to 200 μL. Therefore, for each volume of coating antibodies, optimal concentrations were determined to provide an optical signal of 1.0–1.5, and then they were compared in terms of the sensitivity of ERY determination (Figure 3D). Increasing the surface area of wells coated with antibodies from 100 µL to 200 and 300 µL resulted in a corresponding change in IC_50_ of 0.091, 0.038, 0.042 ng/mL, respectively. The volume of substrate (200 µL) supplemented with the volume of stop solution (50 µL) was the limiting factor, since further volume increases are not applicable for spectrophotometer reading. Therefore, when coated with 300 μL of antibodies, no tendency to increase sensitivity was observed. Thus, with a 200 µL volume of coating antibodies and a maximum possible sample volume of 275 µL, the sensitivity of macrolide detection increased to 0.038 ng/mL (ERY).

### 3.6. Effect of Oriented Coating of Antibody and Competitive Stage Duration on Assay Sensitivity

**Figure 4:**
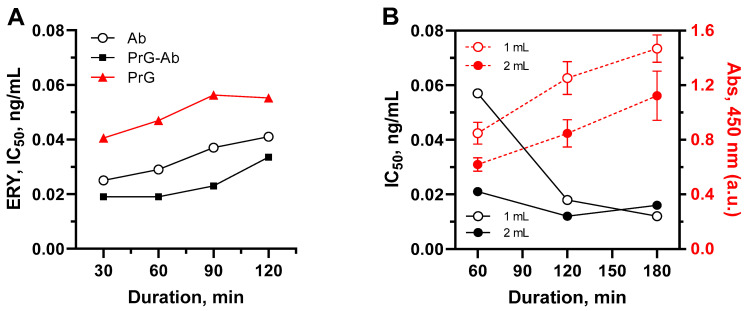
(**A**) Effect of oriented antibody coating and competitive stage duration on assay sensitivity. Ab, coated antibody; PrG, coated protein G in self-assembly assay; PrG-Ab, antibody captured by protein G in step-by-step assay; (**B**) dependence of IBA parameters on the duration of incubation. Each IC_50_ values were calculated from the standard curves generated from respective assay variants using triplicate ERY concentrations. Absorbance values represented by average (n = 3) ± SD.

Another important consideration was the enhancement of the functional activity of antibodies through their oriented immobilization on the surface of polystyrene and its potential impact on the method sensitivity [29]. This was achieved by interacting the adsorbed protein G with the antibody Fc fragments, allowing the antibody-binding fragments to remain spatially accessible. This interaction could be implemented in a step-by-step manner or as a result of self-assembly (Figure 4A).

The step-by-step procedure involved the initial formation of a complex between the coated protein G and the antibody (PrG-Ab), followed by a competitive assay step. An alternative self-assembly method implied the competitive interaction of antibody with an analyte (sample) and a tracer in the solution with simultaneous binding to the coated protein G (PrG).

Improving the functional activity of antibodies through oriented coating made it possible to increase assay sensitivity (Figure 4A, rectangle vs. circle) and reduce the consumption of antibodies by two times. However, the additional PrG-Ab interaction step extended the assay duration by 1 h. The one-step self-assembly assay format (Figure 4A, triangle) turned out to be comparatively insensitive. Self-assembly assumed the simultaneous completion of two interactions PrG–Ab–HRP-aecAZI, which, as a rule, requires a prolonged incubation (2 h) or higher concentrations of reagents to maintain an adequate output signal [30]. Thus, the oriented antibody using stepwise coating provides an additional gain in sensitivity compared to randomly coated antibodies [31].

### 3.7. Effect of Sample Volume Increase on Sensitivity Assessed in IBA and IFA

**Figure 5:**
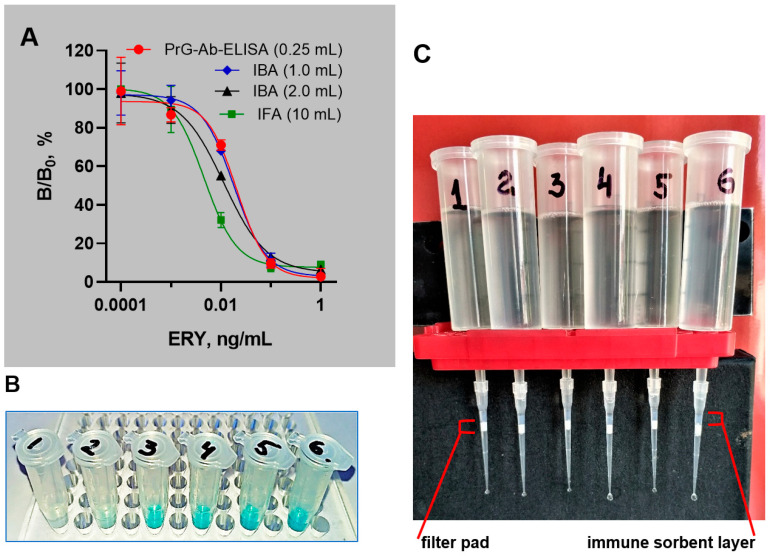
(**A**) Standard curves of ERY determination from different volume samples conducted in PrG-Ab ELISA (250 µL), IBA (1 mL and 2 mL), and IFA (10 mL). Each symbol indicates the average value (*n* = 3), and the error is represented by the standard deviation. (**B**) Visual results of IBA of 1 mL-solutions ERY 1000 pg/mL–0.1 pg/mL, 0 pg/mL (1–6). (**C**) Instillation of 10 mL sample through a microtip with a layer of sorbent on the filter in IFA.

Due to the fact that the plate format does not allow the analysis of samples with a volume of more than 250 μL, the effect of this factor on assay characteristics was further evaluated in IBA and IFA, where the direct competitive immunoassay principle remained unchanged. Instead of a polystyrene plate, we used antibody covalently immobilized on agarose as a solid phase, which remained active when stored at 4 °C for a year. We maintained a constant quantity of the tracer HRP(pi)-aecAZI across the compared assay formats, but optimized the volume of immune beads to achieve an optical signal output in the range of 1.0–1.5. IBA was performed by incubating beads and tracer with 1 and 2 mL samples in Eppendorf tubes for 1–3 h with slow rotation (Figure 5B). Prolonged incubation resulted in a higher output signal, but 2 h was sufficient to reach an adequate absorbance rate when testing both 1 mL and 2 mL samples (Figure 4B). As shown in Figure 5A, sensitivity continued to improve with increasing sample volume.

IFA allowed the analysis of 10 mL samples, which were pre-supplemented with a tracer and freely dropped through a layer of immune sorbent placed in the filtered microtip overnight (Figure 4B and Figure 5C).

Thus, the approach using a heterologous hapten aecAZI was one of the steps taken to improve the assay sensitivity [32]. Subsequently, by utilizing only the internal resource of the prepared reagents without additional labels, enhancers, or amplifiers, the sensitivity of the assay can be significantly increased by a larger sample volume, surpassing the sensitivity of many reported assays (Table 2).

### 3.8. Environmental Water Analysis and Recovery Examination

The high sensitivity achieved through the use of a heterologous hapten and the sample-volume-mediated effects, combined with specificity for several macrolides, enabled the detection of antibiotic pollution in environmental water using an immunoassay. We collected 105 water samples n at 17 geographical points from various depths of Lake Onega and its influents near Petrozavodsk three times a year (September-2022, March-2023, and May-2023) (Table S2). Screening analysis was conducted in parallel using PrG-ELISA and HPLC-MS/MS, which revealed no macrolide contamination.

To determine the recovery of macrolides in spiked blank environmental water samples, we performed PrG-ELISA, using ERY as a standard, and the activity of the samples was expressed in ERY equivalents (Table S3). By using the cross-reactivity ratios of known macrolides relative to ERY, we were able to approximate their concentrations in the samples. The results showed a quite adequate level of recovery (ranging from 71.7 to 141.3%), indicating the suitability of the test for detecting macrolide contamination in natural water.

## 4. Conclusions

Assay parameters, such as sensitivity and LOD, which are typically reported as analyte concentrations, actually depend on the amount of analyte in the assay rather than its concentration. This fundamental fact is often overlooked due to the established protocol of the analysis procedure. In our current study, we used macrolide antibiotics as model analytes to demonstrate that increasing the sample volume represents an effective but underutilized approach for improving the sensitivity of immunoassays. By gradually increasing the sample volume while maintaining the same reagents in direct competitive ELISA, IBA, and IFA formats, we observed significant changes in assay parameters, as summarized in Table 3.

The resulting changes in sensitivity and LOD were remarkable, exceeding 50-fold, highlighting the profound impact of sample volume on assay performance. Additionally, oriented antibody coating through protein G-mediated capturing demonstrated notable improvements in assay sensitivity and reduced antibody consumption.

Furthermore, the synthesis of a new heterologous hapten, aecAZI, contributed to a 5-fold increase in sensitivity and the improved group detection of macrolides due to the better recognition of AZI. Consequently, these assays, characterized by their picogram-level sensitivity and group recognition of ERY, CLA, ROX, AZI, ESE, and DIR, could be suitable for monitoring macrolide pollution in the environment. We conducted analyses on a series of natural water samples from Lake Onega and its influents near Petrozavodsk from 2022 to 2023 using both the developed immunoassay and HPLC-MS/MS and revealed no macrolide antibiotic contamination. The suitability of the test for detecting the macrolide contamination in natural water was further confirmed by the recovery of macrolides from spiked blank samples, ranging from 71.7 to 141.3%.

## Figures and Tables

**Table 1:** The cross-reactivity (CR) of anti-BSA-cmoCLA(ae) antibodies with macrolides (MLs).

MLs	GEL- cmoCLA(ae) *		OVA-cmoERY(ae)		OVA- aecAZI(ga)		OVA-aecAZI(dma)		OVA(pi)- aecAZI	
	IC_50_, ng/mL	CR,%	IC_50_, ng/mL	CR,%	IC_50_, ng/mL	CR,%	IC_50_, ng/mL	CR,%	IC_50_, ng/mL	CR,%
CLA	0.16	100	0.172	100	0.043	100	0.157	100	0.044	100
ERY	0.20	68.2	0.240	71.7	0.049	87.8	0.223	70.4	0.042	105
ESE	-	-	0.466	36.9	0.094	45.7	0.352	44.6	0.074	59.5
ROX	0.14	125	0.125	137.6	0.040	107.5	0.157	100	0.051	86.3
AZI	1.63	8.8	1.990	8.6	0.315	13.7	1.220	12.9	0.107	41.1
DIR	-	-	0.554	31.0	0.136	31.6	0.544	28.9	0.096	45.8
TUL	-	-	629.9	2.7	134.8	0.03	379.5	0.04	5.403	0.81
OLE	-	-	75.75	0.2	40.10	0.11	287.2	0.05	45.64	0.1

* The CR data were recalculated from those presented in [18]. Dash means that the compound has not been examined.

**Table 2:** Comparable characteristics of reported immunoassays for macrolide determination.

Assay (Matrix)	Immunogen	Antigen	IC_50_//LOD, ng/mL	CR, %		Reference
ICA (Milk)	Commercial	Commercial	ni//5	ERY	100	[33]
FM-ICA (Milk)	Commercial	Commercial	ni//0.13	ERY	100	[34]
IC-ELISA (Milk)	BSA-cmoERY(ma)	OVA-cmoERY(ae)	0.94//0.3	CLA ERY ESE ROX AZI DIR	26.7 100 - 14.8 0.9 157	[19]
ICA (Milk)	BSA-cmoCLA(ae)	OVA-cmoCLA(ae)	0.16//0.095	CLA ERY ESE ROX AZI DIR	100 30.1 - 21.1 16.2 -	[35]
LFIA (Breast milk)	BSA-cmoCLA(ae)	GEL- cmoCLA(ae)	0.45//0.12	CLA ERY ROX DIR AZI	100 7.5 97.8 5.4 5.7	[20]
FPIA (Milk)	BSA-cmoERY(ma)	OVA-cmoERY(ae)	7.4//14.1	CLA ERY ESE ROX AZI DIR	26.7 100 43.7 92 - 157	[36]
IC-ELISA (Water) IFA (Water)	BSA-cmoCLA(ae)	OVA(pi)-aecAZI HRP(pi)-aecAZI	0.04//0.01 0.005//0.001	CLA ERY ESE ROX AZI DIR	100 105 59.5 86.3 41.1 45.8	Present study

IC-ELISA—indirect competitive ELISA; ICA—immunogold chromatographic assay; FM-ICA—fluorescent microsphere-based immunochromatographic assay; LFIA—latex lateral flow immunoassay; FPIA—fluorescence polarization immunoassay; IFA—immunofiltration assay; ni—not indicated; ma—mixed anhydride method.

**Table 3:** ERY immunodetection parameters depending on the volume of the test sample.

Assay Format, Competitive Step Duration	Coating Ab Volume, µL	Standard/Sample, µL	IC_50_, ng/mL	IC_20_-IC_80_, ng/mL	IC_10_, ng/mL
ELISA, 1 h	100	25	0.31	0.097–0.907	0.04
	100	50	0.23	0.085–0.565	0.04
	100	100	0.14	0.037–0.400	0.008
	100	200	0.098	0.029–0.253	0.011
	100	275	0.091	0.043–0.184	0.024
	200	275	0.034	0.008–0.119	0.002
	300	275	0.040	0.012–0.119	0.006
PrG-ELISA, 1.5 h	200	250	0.055	0.015–0.199	0.007
PrG-Ab-ELISA, 0.5 h	200	250	0.020	0.006–0.054	0.002
IBA, 2 h	10	1000	0.018	0.006–0.056	0.003
IBA, 2 h	20	2000	0.012	0.003–0.057	0.001
IFA, overnight	50	10,000	0.005	0.002–0.019	0.001

## Data Availability

All original data in this study are included in the article and supporting information.

## Supplementary Materials

The following supporting information can be downloaded at: https://www.mdpi.com/article/10.3390/bios13100921/s1, Methods: HPLC-MS/MS procedure; Methods: SPE procedure; Figure S1: Formulas of macrolide antibiotics used in research; Figure S2: Chromatogram of macrolides by HPLC-MS/MS; Table S1: HPLC-MS/MS parameters of macrolide family antibiotics; Table S2: Environmental water samples listing; Table S3: Recovery macrolides from spiked environmental water using PrG-ELISA.
