# Supplementary material for: Immunotechniques for the Group Determination of Macrolide Antibiotics Traces in the Environment Using a Volume-Mediated Sensitivity Enhancement Strategy
Source: Biosensors (Basel). 2023 Oct 10;13(10):921. doi: 10.3390/bios13100921 (PMC10605010; doi:10.3390/bios13100921)
Supplement: Supplementary file 1 [file biosensors-13-00921-s001.zip › biosensors-2623201-supplementary.pdf]

Supplementary Information

Immunotechniques for the Group Determination of Macrolide Antibiotics Traces in the Environment Using a Volume-Mediated Sensitivity Enhancement Strategy

Figure S1. Formulas of macrolide antibiotics used in research ………………………………2

HPLC-MS/MS procedure …………………………………………………………………3

SPE procedure ……………………………………………………………………………..3

Table S1. HPLC-MS/MS parameters of macrolide family antibiotics………………………...4

Figure S2. Chromatogram of macrolides by HPLC-MS/MS…………………………………..5

Table S2. Environmental water samples listing ……………………………………………7

Table S3. Recovery macrolides from spiked environmental water using PrG-ELISA……..…8

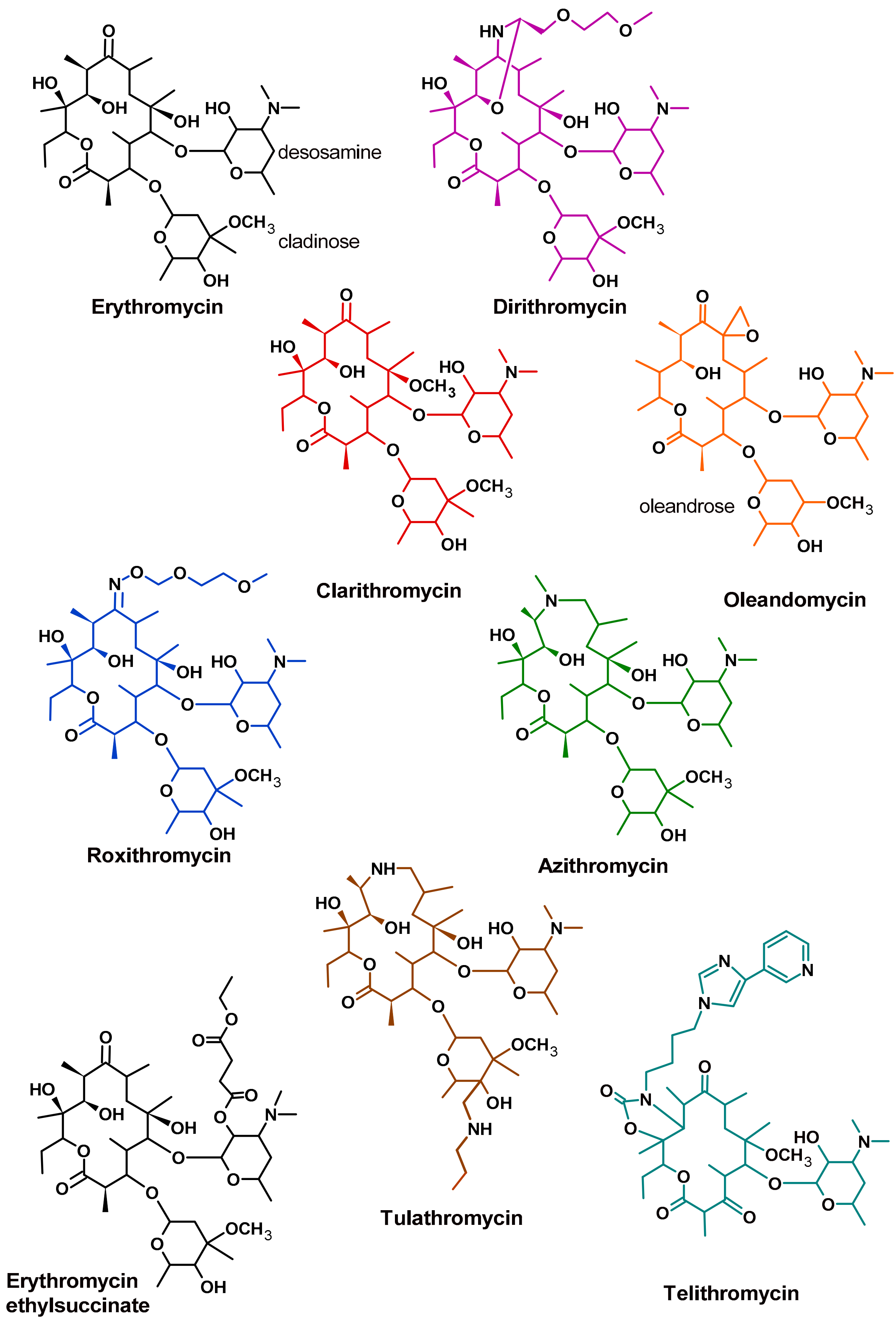

Figure S1. Formulas of macrolide antibiotics used in research.

HPLC-MS/MS procedure

A Shimadzu HPLC Nexera X2 liquid chromatograph equipped with a binary pump and an autosampler was used. Separation was carried out using an Acclaim™ 120 C18 column (100 × 2.1 mm) with an adsorbent grain size of 3.0 μm (Thermo Scientific, United States) in a gradient elution mode. The column oven and the autosampler were maintained at 40 and 15 ◦C respectively during operation. The analytes were separated using a mobile phase containing 0.5% acetic acid in water (eluent A) and 0.5% acetic acid in mixture ACN / MeOH 1:1 (eluent B). The gradient program was as follows: 0–1.0 min 5% B; 1.0 –8.0 min: increase from 5% to 95% B; 8.0–9.0 min: 95% B; 9.0–9.2 min: return to 5% B; 9.2–10.0 min: 5% B. The mobile phase flow rate was 0.3 mL min− 1. The injection volume was 10 μL. A triple quadrupole mass spectrometer LCMS 8060 Shimadzu was configured to collect data in the multiple reaction monitoring (MRM) mode.

The following optimal parameters for ESI were set: nebulizer gas: 3 L/min, drying gas: 10 L/min, heating gas: 10 L/min, interface temperature: 300 °C, desolvation line temperature: 250 °C. Multiple reaction monitoring (MRM) conditions, collision energy (CE), Q1 Pre Bias (V), Q3 Pre Bias (V) were first optimized for each analyte by injecting solutions of the macrolide antibiotic standards prepared in the mobile phase. Characteristic molecular ions were selected as precursor ions, and two product ions were monitored for each compound. For quantification, the most intense MRM transition was monitored, along with a second transition for identity confirmation. MRM parameters and retention times for macrolides and internal standards are provided in Table S1.

SPE procedure

SPE cartridges (Oasis HLB) were preconditioned with methanol (3 mL) and then with Milli-Q water (3 mL). 10 ml sample were passed through the cartridges and then the cartridges were rinsed with Milli-Q water once (3 mL) and dried under vacuum for 10 min to remove excess water. Then, the analytes were eluted with 3 mL of MeOH. The eluates were evaporated under a gentle stream of high purity nitrogen at 40 °C until they were almost dry, and then re-dissolved in 100 μL of 5 % mobile phase B.

Table S1. HPLC-MS/MS parameters of macrolide family antibiotics

Compound	Ionization mode	m/z of precursor ion	m/z of product ions	Q1 Pre Bias (V)	Collision energy (V)	Q3 Pre Bias (V)	Retention time, min²
Tylvalosine	ES+	1042,4	174,2*	-30	-43	-18	7,7
			229,2	-50	-39	-15	
Tulathromycin	ES+	806,4	577,3*	-22	-27	-20	5,4
			158,1	-22	-41	-16	
Spiramycin	ES+	422,4	101,1*	-20	-21	-10	5,7
			142,2	-20	-16	-14	
Clarithromycin	ES+	748,5	158,1*	-22	-33	-27	7,4
			590,4	-36	-22	-30	
Tylosin	ES+	916,3	174,2*	10	-26	-39	6,9
			772,3	10	-26	-31	
Tilmicosin	ES+	869,4	174,2*	-24	-46	-17	6,2
			696,3	-24	-42	-26	
Erythromycin	ES+	734,3	158,1*	-20	-32	-16	6,8
			576,2	-20	-21	-28	
Oleandomycin	ES+	814,5	158,2*	-22	-25	-17	7,2
			116,1	-22	-44	-22	
Azithromycin	ES+	749,5	591,4*	-20	-31	-22	5,8
			158,2	-20	-37	-10	
Tildipirosin	ES+	734,5	561,3*	-20	-33	-20	5,7
			174,3	-20	-39	-11	
Dirithromycin	ES+	835,6	158,1*	-22	-38	-16	6,1
			677,6	-22	-26	-38	
Telithromycin	ES+	812,5	655,3*	-22	-32	-24	5,8
			623,2	-24	-44	-22	
Midecamycin	ES+	814,5	201,3*	-22	-30	-21	7,2
			614,3	-24	-27	-22	
Roxithromycin	ES+	837,5	158,2*	-24	-34	-16	7,5
			116,2	-24	-43	-11	
Erythromycin ethylsuccinate	ES+	862,5	286,1*	-24	-30	-13	7,6
			129,0	-24	-43	-12	
(IS) Azithromycin-d3	ES+	752,4	594,4	-22	-31	-22	5,8
(IS) Roxitromycin-d7	ES+	844,6	686,5	-24	-23	-26	7,5

*Quantitation ion

(IS) Internal standard

Tylvalosine

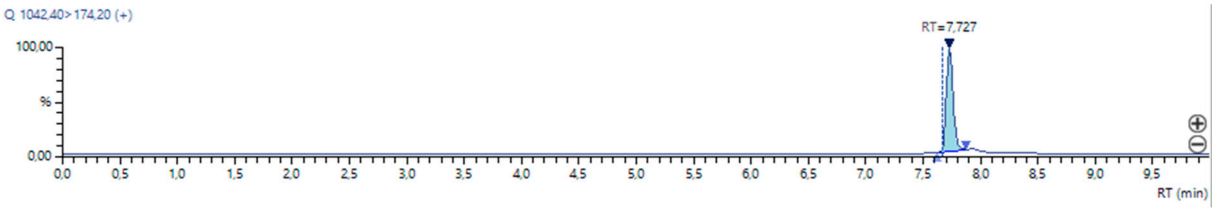

Tulathromycin

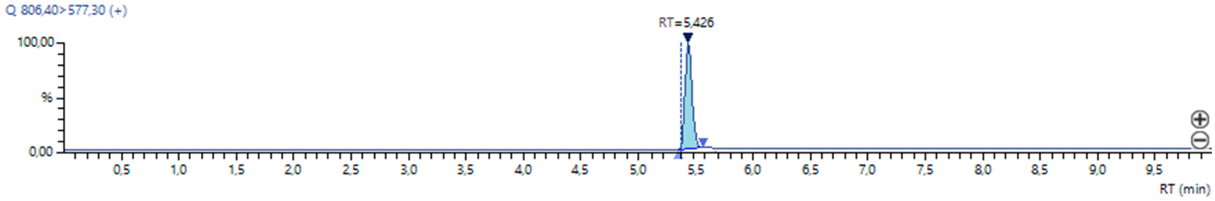

Spiramycin

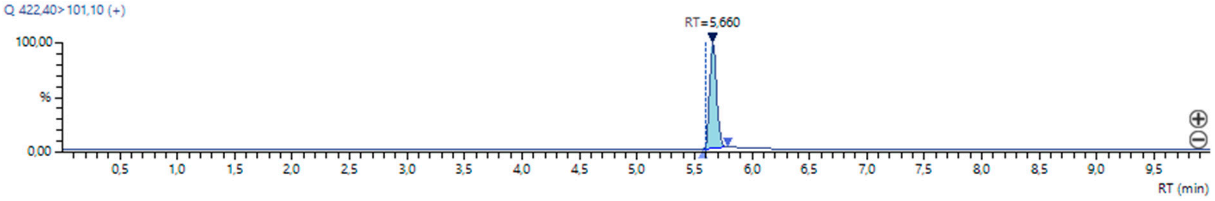

Clarithromycin

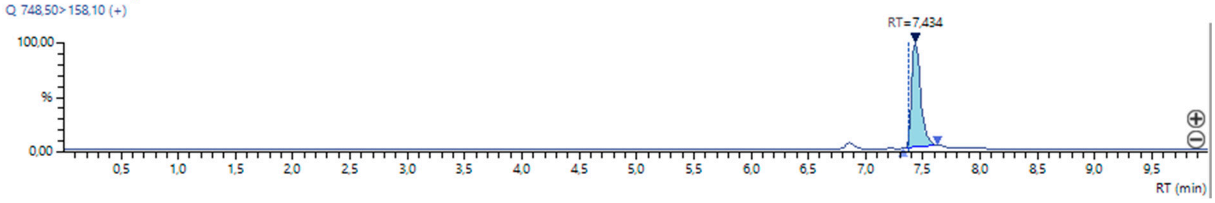

Tylosin

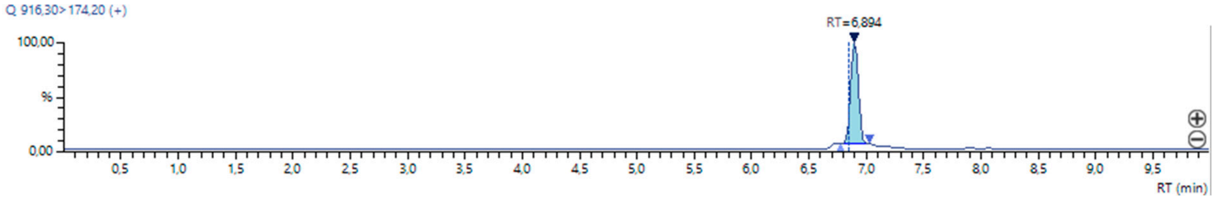

Tilmicosin

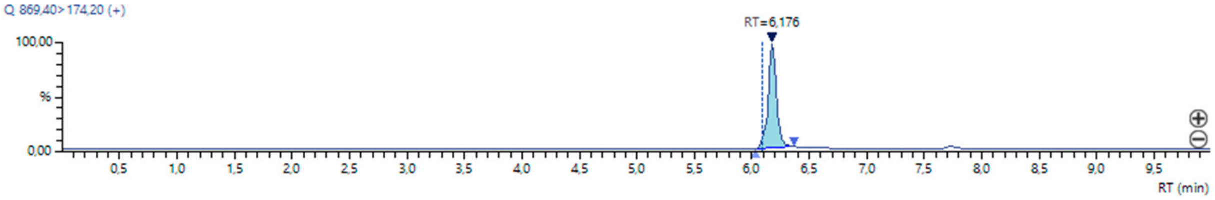

Erythromycin

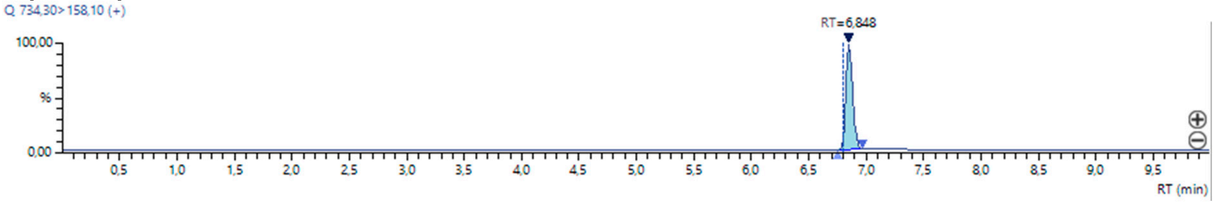

Oleandomycin

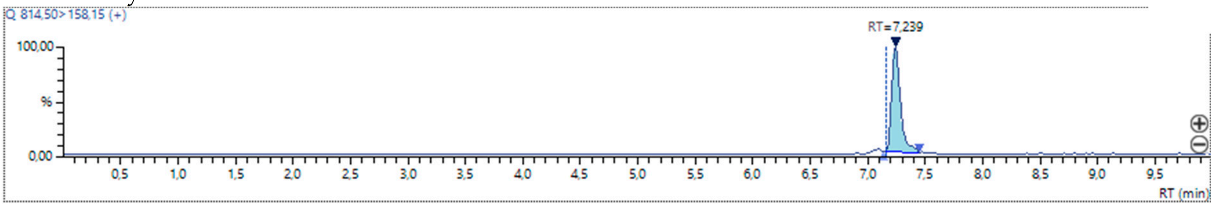

Azithromycin

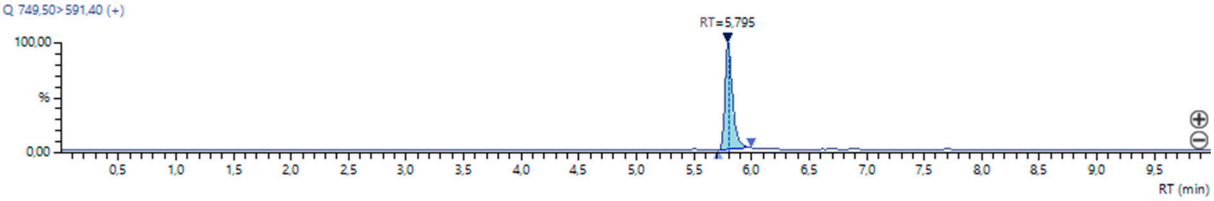

Tildipirosin

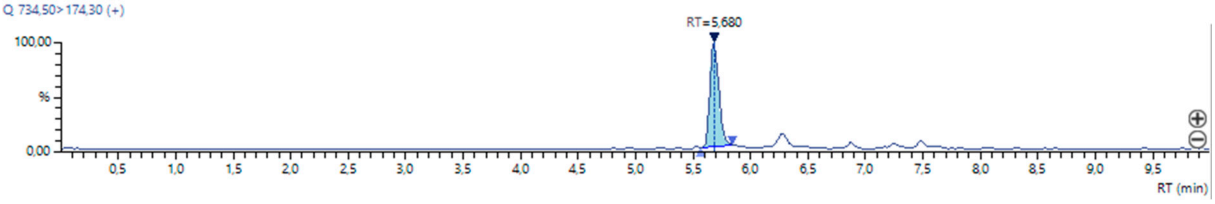

Dirithromycin

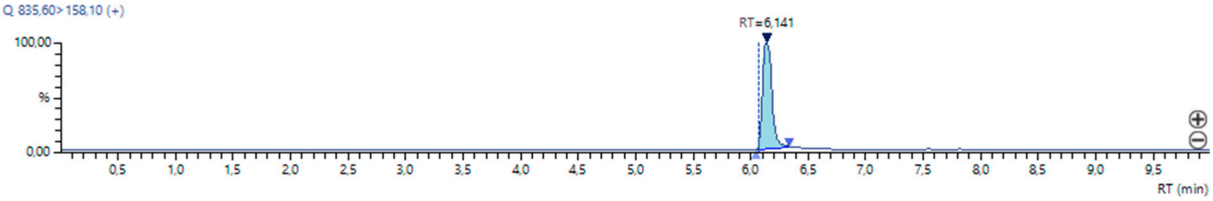

Telithromycin

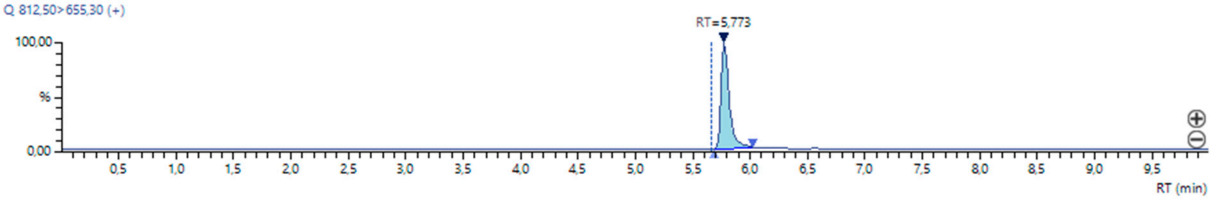

Midecamycin

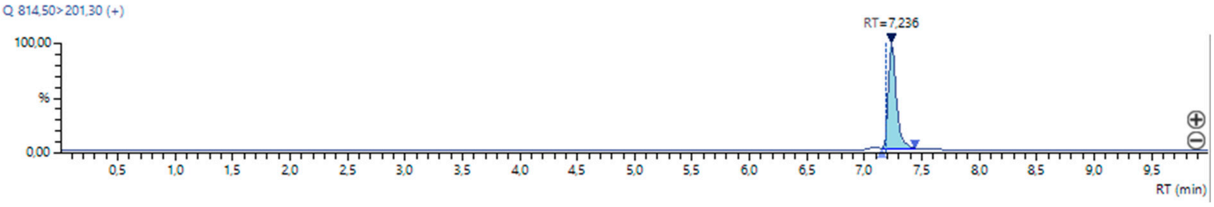

Roxithromycin

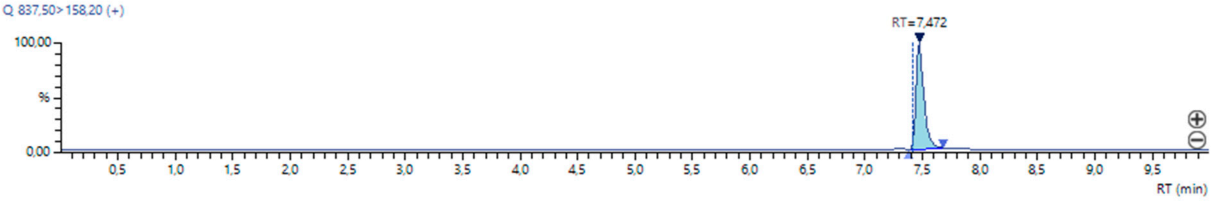

Erythromycin ethylsuccinate

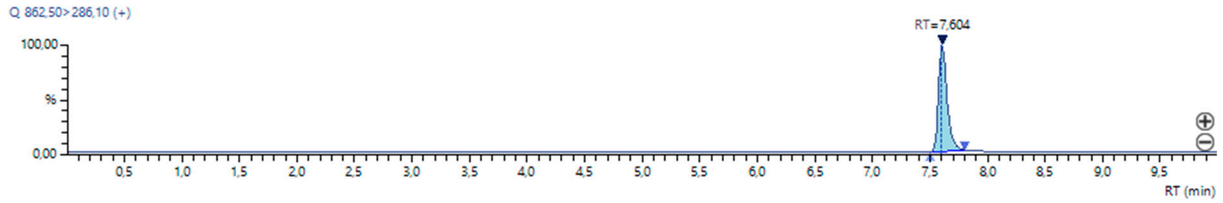

Figure S2. Chromatogram of macrolides by HPLC-MS/MS

Table S2. Environmental water samples listing

Geographical coordinates		Depth, m	Sample number, collection time		
N, degree	E, degree		September-22	March-23	May-23
62.01544444	34.6865	0.5	#127		#206
		20	#128		#207
		32	#129		#208
62.07566667	34.47463889	0.5	#130	#171	#209
		10	#131	#172	#210
		30	#132	#173	#211
		50	#133	#174	#212
		65	#134	#175	#213
62.10136111	34.40647222	0.5	#135	#176	#214
		10	#136	#177	#215
		20	#137	#178	#216
		28	#138	#179	#217
		34	#139	#180	#218
62.18316667	34.25666667	0.5	#140	#192	#229
		3	#141	#193	#230
		7	#142	#194	#231
		10	#143	#195	#232
62.16736111	34.27638889	1	#144	#187	#224
		4	#145	#188	#225
		7	#146	#189	#226
		10	#147	#190	#227
		13	#148	#191	#228
62.13583333	34.31044444	1	#149	#181	#219
		5	#150	#182	#220
		10	#151	#183	#221
		18	#152	#184	#222
		26	#153	#185	#223

62.08494444	34.37252778	1	#154	#168	
		23	#155	#169	
62.07288889	34.39002778	0.5	#156	#162	#235
		15	#157	#163	
		25	#158	#164	
		33	#159	#165	#238
62.06533333	34.40588889	0.5	#160	#166	
		15.5	#161	#167	
61.83474	34.35986667	0.5		#170	
62.20609	34.28452222	0.5		#196	
62.10792	34.26927778	0.5		#197	
61.676	34.56022222	0.5		#198	
61.72972	34.46936111	0.5		#199	
61.78697	34.39544444	0.5		#200	
61.79792	34.3685	0.5		#201	
61.918882	34.83359	0.5			#241
		39			#242

Table S3. Recovery macrolides from spiked environmental water samples using PrG-ELISA

Macrolide	Spike level, ng/mL	Sample dilution	Found concentration, ERY eqs, ng/mL	CR coefficient recalculation	RC, %
ROX	12.5	100	7.367	8.96	71.7
AZI	3.33	100	1.27	3.17	95.2
CLA	2.00	100	1.97	2.07	103.5
ESE	1.05	10	0.50	0.88	83.8
DIR	0.75	10	0.37	0.857	114.3
AZI	0.15	10	0.083	0.212	141.3
CLA	0.075	10	0.069	0.073	97.3
